# Living with muscular dystrophy: health related quality of life consequences for children and adults

**DOI:** 10.1186/1477-7525-5-31

**Published:** 2007-06-06

**Authors:** Martha A Grootenhuis, Judith de Boone, Anneke J van der Kooi

**Affiliations:** 1 Pediatric Psychosocial department, Emma Children's Hospital AMC, Amsterdam, P.O. box 22700, 1100 DE, Amsterdam, The Netherlands; 2 Rehabilitation Center "de Trappenberg", Huizen, The Netherlands; 3 Department of Neurology, Academic Medical Center, University of Amsterdam, The Netherlands

## Abstract

**Background:**

Muscular dystrophies are chronic diseases manifesting with progressive muscle weakness leading to decreasing activities and participation. To understand the impact on daily life, it is important to determine patients' quality of life.

**Objective:**

To investigate Health Related Quality of Life (HRQoL) of children and adults with muscular dystrophy (MD), and to study the influence of type and severity of MD on HRQoL in adult patients.

**Methods:**

Age-related HRQoL questionnaires were administered to 40 children (8–17 years), and 67 adult patients with muscular dystrophies.

**Results:**

Significant differences in HRQoL were found in children and adults with MD compared to healthy controls. Patients with Becker muscular dystrophy reported a better HRQoL on the several scales compared to patients with other MDs. Severity was associated with worse fine motor functioning and social functioning in adult patients.

**Conclusion:**

This is one of the first studies describing HRQoL of patients with MD using validated instruments in different age groups. The results indicate that having MD negatively influences the HRQoL on several domains.

## Background

Duchenne muscular dystrophy (DMD) is the most frequent muscular dystrophy of childhood [1]. Disease progression is severe, leading to loss of ambulation around the age of 10 and respiratory insufficiency necessitating night-time ventilatory support around the age of 20 years [1]. Becker muscular dystrophy (BMD) runs a more variable and usually more benign course. Sarcoglycanopathies (limb girdle muscular dystrophies (LGMD) 2C-F), can have a DMD or BMD like course [2]. Cardiac involvement can be prominent in these disorders [3,4]. LGMD2A usually starts in childhood and leads to loss of ambulation around the fourth decade. No cardiac involvement is encountered in this disorder [2]. Both dystrophinopathies and limb girdle muscular dystrophies are chronic and progressive diseases which lead to decreasing activities and participation. Patients will have to learn to cope with declining motor abilities, which influence their quality of life (QoL). QoL is a multidimensional structure and contains generally three general domains: physical, psychological and social functioning of a patient [5]. In the study of Bothwell [6] it was shown that families with a son diagnosed with DMD considered QoL as more important above physical functioning. In addition, various studies have shown that healthcare professionals underestimate the QoL of their patients, which may affect the level of care the patients receive [7,8].

QoL has been studied mostly in end of life care issues [6-8]. Some studies have been performed to identify the manner in which individuals with MD perceive their QoL in their current living situation, but many studies lack the administration of standardized QoL instruments. An example is a recent study by Rahbek et al. [9] in which adults with DMD were interviewed. They conclude that the patients report their quality of life to be excellent. Problems were reported in the area of qualifying education, love life and pain. Several others report negative consequences in the areas of occupation, energy or emotional experiences [10,11]. QoL has been objective of many studies among patients with chronic diseases, in which health status, i.e. the possibility of an individual to perform his daily activities and to fulfil his needs, has been the major perspective. Functional health and Health Related QoL are often used as outcome measures to evaluate patient's well being. The terms are often mixed up in literature. Functional health is defined as an individual's ability to perform normal daily activities, essential in order to meet basic needs, to fulfil usual roles, and to maintain health and well-being. Quality of life (QoL) is defined as an individual's perception of their position in life, in the context of the culture and value systems in which they live, and in relation to their goals, expectations, standards and concerns. HRQoL is defined as QoL in which a dimension of personal judgement over one's health and disease is added. [12] In case of children (HR)QoL is influenced also by factors such as the ability to participate in peer groups and the ability to keep up with developmental activities. Furthermore, it is important not to rate health problems only, but also to include the impact of these problems as perceived by the person involved. Such studies have not been done often yet.

Measurement in children requires age-adjusted questionnaires because children need adequate language skills and the cognitive ability to interpret the questions. Unfortunately reliable and valid translated HRQoL instruments are still scarce in the pediatric field [12]. Consequently, parents usually function as the major informants in pediatric assessments, especially in young children. However, self-reported quality of life is considered as most informative and important [12].

The present study aimed to answer the following three questions: (1) Does HRQoL of children with MD, measured with self-reports, differ from that of a group of healthy children? (2) Does the HRQoL of adults with MD differ from that of a group of healthy controls? (3) Are type of MD and severity predictors of HRQoL of adults with MD? Based on previous research with children and adults with chronic diseases we hypothesize that HRQoL will be affected on all domains.

## Methods

### Participants

Between 2001 and May 2005 patients with Duchenne muscular dystrophy, Becker muscular dystrophy, female carriers of both diseases and patients with a sarcoglycanopathy were asked to participate in an ungoing study. Tertiary Dutch neuromuscular referral centres in academic hospitals or rehabilitation centres contacted most patients. Inclusion criteria for eligibility were the following: age 8 to 80 years, genetic confirmation of DMD, BMD, carriership or sarcoglycanopathy by DNA analysis or muscle biopsy demonstrating absent, reduced or abnormal dystrophin or sarcogycan on immunohistochemical and/or immunobiochemical analysis. Carriers were considered symptomatic when symptoms attributable to skeletal muscles, like cramps or muscle weakness, were present. Patients or carriers who recently had a cardiovascular event or underwent a major surgical intervention were excluded. For the present study we approached patients from our own clinic who were included between May 2002 and May 2003. In addition, calpainopathy (LGMD2A) patients who were identified in our large LGMD survey and in whom mutations in the calpain-3 gene were demonstrated [13] were asked for their cooperation. All patients received an introduction letter, in which the aim of the study was explained and participation was requested. After permission was given, data were collected with questionnaires. The interviews were mostly done by phone. Some were done in the hospital or in the patients' home. All interviews, with both pediatric as well as adult patients, were done by one medical student (JB). In all cases, the interviewer read out aloud the question and all possible answers and subsequently, the patient could choose one of the answers. In case of pediatric patients, parents were asked to leave to room to give the child the opportunity to answer questions openly. At the end of the interview the patient was encouraged to give his/her comment on the questionnaire. Several clinical characteristics were extracted from the medical files, including type of diagnosis, severity of disease and the child's intelligence. In the adult population type and severity of muscle dystrophy were determined. Severity was judged with Brooke's scale, a functional index for use of legs and hip muscles (ranging from 1 = walking and climbing stairs without help to 10 = bedridden). The cut off value for severity (yes/no) in adults was the loss of independent walking (Brooke score ≥ 5) [13,14].

### Instruments

The Dutch Institute of Prevention and Health and the Leiden University Hospital (TNO-AZL) designed questionnaires for measuring HRQoL for different age groups: the TNO-AZL Children's Quality of Life questionnaire (TACQoL) for children ages 6–15 years [15-18], and the TNO-AZL Adult Quality of Life questionnaire (TAAQoL) for 16 years and older [19]. These questionnaires are generic Dutch instruments that measure health status problems weighted by the impact of the health status problems on well-being. It offers the respondent the possibility of differentiating between their functioning and the way they feel about it. Most of the items consist of two questions linked to one another. On the first one the respondent can rate whether or not a specific problem occurred in the past few weeks. The second one is about the possible negative emotional responses to the problems. The respondent can indicate how he felt about this problem on a four point Likert scale: fine – not so good – quite bad – bad. The items are clustered into multi-item scales with higher scores indicating higher quality of life.

### TACQOL: children

**Figure 1:**
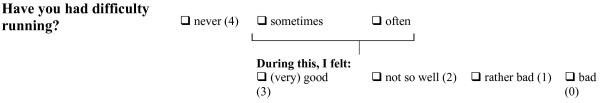
An example of a TACQol-CF question translated from the Dutch original.

The TACQoL was developed and validated in a large sample of Dutch school-going children aged 8–15 years, including children with or without a chronic medical condition [17,18]. Data were collected in twelve municipal health services located throughout the Netherlands. The *TACQOL *assesses functioning of children aged *8–15 *years with the Child-Form (CF). The instrument contains seven scales of eight items each: physical functioning (e.g. the child is experiencing stomach-aches or abdominal pain, feeling sleepy), autonomy (e.g. is having difficulties going to school alone, or doing hobbies independently), motor functioning (e.g. problems running, or with balance), cognitive functioning (e.g. difficulties paying attention or concentrating, difficulty writing) and social functioning (e.g. impaired ability to play or talk with other children, or to feel at ease with other children), positive emotions (e.g. feelings of joy or contentedness) and negative emotions (e.g. sadness or aggression). A concretely and specifically formulated health status problem, if reported, leads to a question about the emotional response. Figure 1 shows an example of such a question. On each item of the first five domains the respondent indicates the extend to which a specific problem occurred in the past few weeks (never (4) – sometimes- often). If a problem occurred, the child can indicate how he/she felt about this problem on a four point Likert scale: (very) good (3) – not so well (2) – rather bad (1) – bad (0.) The emotional reaction to the complaints represents the 'Health-Related' component, which is reported here. Scores for these domains range from 0–32. On the domains regarding positive and negative emotions, respondents can indicate on a three-point Likert scale whether the presented feelings were present in recent weeks (never (2)-occasionally (1) -often (0)). Scores on these two domains range from 0–16. Numbers between brackets in Figure 1 refer to the values resulting in the HRQoL scores. It takes approximately 15–20 minutes to fill in the questionnaire. In the calculation of the scale scores one or two missing combined-item scores are allowed for. They are replaced by the mean value of the non-missing (combined-) item scores. For respondents with more missing combined item scores per scale, the scale score is assumed to be missing.

Scale structure and reliability proved less satisfactory for the older children between 12–15 years. Therefore it was advised to adapt the scale structure for the older children by removing some items for the original social scale and one scale in its totality (autonomy). Since children with chronic diseases are treated by the pediatrician until the age of 18, patients in our population aged 15–17 years were also administered the TACQoL-CF.

### TAAQoL: adults

The TAAQoL [19] is a multidimensional instrument, with 12 scales: gross motor functioning (e.g. difficulty walking, bending), fine motor functioning (e.g. difficulty cutting papers or opening a can), cognitive functioning (e.g. difficulty remembering or concentrating), sleep (e.g. sleeping restlessly, lay awake a lot), pain (e.g. back-ache, pain in neck-shoulders), social functioning (e.g. talk to others; visit friends), limitations of daily activities (e.g. difficulties with work; done less work), sexual functioning (e.g. had less sex), vitality (e.g. feel energetic; tired), happiness (e.g. feel joyful; cheerful), depressive moods (e.g. feel sad or worried) and aggressiveness (e.g. feel angry; aggressive). In all scales, except in the scales concerning sexual functioning (two questions) and aggressiveness (3 questions), each scale consists of four questions. It takes approximately 10–20 minutes to fill in the questionnaire. For the TAAQoL, the scale scores are obtained by adding item scores within scales and transforming crude scale scores to a 0–100 scale. In the calculation of the scale scores one missing combined-item score per scale is allowed for. A missing item score is replaced by the mean value of the non-missing (combined-) item scores. For respondents with more missing combined-item scores per scale, the scale score is assumed to be missing. Patients older than 18 years were administered the TAAQoL. Norm data from the general Dutch population were available. The reference study was carried out in two samples of the general population. Several steps were undertaken to study the psychometric aspects of the questionnaire. E.g. the convergent validity was assessed by calculating correlation coefficients with the Dutch versions of the SF-36 and with the "Psychological complaints-scale" of the Hopkins Symptom Checklist. Criterion validity was assessed by testing the differences in scales scores of people with and without chronic conditions and those who visited a doctor versus those who did not during the last 6 months. Based on these results it is concluded that the instrument measure HRQOL on group level in a reliable and valid way [19].

### Statistics

The Statistical Package for Social Sciences (SPSS), Windows version 11.5, was used for all analyses. Before conducting the final analyses several preparation analyses were conducted. Firstly, scales were constructed and missing data imputed on the basis of the guidelines of the questionnaires used. Secondly, the reliability of these scales was calculated. Thirdly, descriptive statistics were used to describe the demographic and medical characteristics of the participants.

#### Children

Normative data for the TACQoL are presented in two age groups: children 8 – 11 years old, and adolescents 12 – 15 years. Since the MD population included children ranging from 8 – 17 years old, it was first tested whether the MD age group 12–15 years (n = 14) differed from the MD age group of 16 and 17 years old (n = 7) with respect to HRQoL. Mann-Whitney U tests revealed HRQoL scales scores to be similar for the two groups, so in further analyses the two groups were taken together. Thereafter, one-sample t-tests were used to compare the means on the TACQoL scales of the children aged 8–11 years with MD with available norms, and for adolescents with that of available norm data. The autonomy and social functioning scales of children aged 12–15 years were not included in the analysis due to a low reliability of those scales in this population. Effect sizes (d) were calculated by dividing the difference in mean score between the patient group and the norm group by the standard deviation of the scores in the norm group. Effect sizes give insight into the magnitude of a difference between independent groups. According to Cohen effect sizes of up to 0.2 were considered to be small, effect sizes of about 0.5 to be moderate and effect sizes of about 0.8 to be large [20].

#### Adults

For the adults with MD, comparisons were made with the data from the general population (n = 4410) of all ages using one-sample t-tests, according to gender [19] and effect sizes were calculated. Differences on all twelve HRQoL mean scale scores between adults with different disease types were examined using analyses of variance (ANOVA) and post-hoc procedures according to Scheffe. Considering the number of Duchenne patients (n = 4), they were left out of the comparison on disease type. To examine differences in HRQoL between adult patients with different severity independent-samples t-tests were used.

Considering the explorative nature of the study p-values of p < 0.05 were considered significant.

## Results

### Patient characteristics

In total 43 children with a muscular dystrophy were approached to participate in the study, of these 40 children agreed to participate (response rate 93 %). Sixty-eight adult patients were approached to participate in the study, and 67 agreed (response rate 99%). Table 1 shows the characteristics of the participating children and adults. From the children five were diagnosed with cardiomyopathy, and from the adult population nine patients. Not all the patients were diagnosed yet at the time of participation in the quality of life study.

**Table 1:** Socio-demographic and medical characteristics of the patients

	Children (n = 40)		Adults (n = 67)	
*Age at study *(years)				
Mean (SD)	12.6 (2.8)		40.7 (13.9)	
Range	8–17		18–67	
*Gender *(%)				
- male	38 (95)		46 (69)	
- female	2 (5)		21 (31)	
*Age categories (%)*				
- 6–11 years	18 (45)			
- 12–17 years	22 (55)			
*Type of disease*	n	%	n *(less severe/severe)*	%
- Duchenne Muscular dystrophy (DMD)	36	90	4 (-/4)	6
- Becker muscular dystrophy (BMD)	-		25 (16/9)	37
- Sarcoglycanopathies (LGMD2C-F)	4	10	15 (2/13)	22
- Calpaïnopathies (LGMD2A)			23 (7/16)	34

Severity: severe = loss of independent walking (Brooke score ≥ 5)

### HRQoL of children and adolescents with MD

The HRQoL of the children aged 8–11 years with MD appeared to be significantly worse (p < 0.05) than the HRQoL of age-matched children from the general Dutch population, on four out of seven scales of the TACQoL: motor functioning, social functioning, positive emotions and autonomy (see Table 2). Surprisingly for physical symptoms a better HRQOL was reported by the children. The differences were moderate for physical symptoms and large for motor functioning, autonomy, social functioning and positive emotions.

**Table 2:** Mean health-related quality of life scores (TACQoL) for children age 8–11 with MD and healthy children

	Children		
	MD	Healthy children	
	Mean (SD)	Mean (SD)	
	N = 18	N = 1094	d
Physical symptoms	26.8 (3.0) *	24.9 (5.1)	-0.4
Motor functioning	20.4 (6.3) ***	29.8 (3.2)	2.9
Autonomy	23.8 (5.8) ***	31.2 (1.9)	3.8
Cognitive functioning	26.5 (4.5)	28.4 (3.9)	0.5
Social functioning	26.7 (4.5) *	29.7 (2.8)	1.1
Positive emotions	11.2 (3.5) *	13.6 (2.5)	1.0
Negative emotions	11.3 (3.0)	11.6 (2.7)	0.1

Higher scores indicate a better quality of life.

d = effect size

One-sample t-test * p < 0.05; ** p < 0.01; *** p < 0.001

Significant differences on the TACQoL-CF between adolescents aged 12–17 years with MD and the norm group were found for the scales of physical functioning and motor functioning (see Table 3). Adolescents reported better physical functioning, but worse motor functioning. These differences were moderate for physical symptoms and large for motor functioning.

**Table 3:** Mean health-related quality of life scores (TACQoL) for adolescents age 12–17 with MD and healthy children

	MD	healthy children	
	Mean (SD)	Mean (SD)	
	N = 22	N = 1249	d
Physical symptoms	26.8 (4.4.)**	23.7 (5.4)	-0.6
Motor functioning	23.2 (4.4)***	29.8 (3.3)	2
Cognitive function	28.1 (4.7)	27.6 (4.1)	-0.1
Positive emotions	12.9 (2.3)	13.0 (2.8)	0.0
Negative emotions	12.4 (2.3)	11.6 (2.6)	-0.3

Higher scores indicate a better quality of life

d = effect size

One sample t-test ** p < 0.01; *** p < 0.00

### HRQoL of adults with MD

Table 4 presents the mean scores on all TAAQoL scales of adults (males and females) with MD in comparison with TAAQoL scores of the general Dutch population (males and females). For both male and female patients a significantly worse HRQoL was found compared to healthy controls on the scales: gross and fine motor functioning, daily activities, vitality, and depressive moods. Males also report problems in the area of pain and aggressiveness, whereas females report problems for sleeping. Neither males nor females report problems on the scales of cognitive and sexual functioning and on the scale of happiness. Surprisingly, a significant difference for females was found for social functioning. Female patients reported a better social functioning.

**Table 4:** Mean scores, SD's and differences by gender between adult patients and comparison group on the scales of the TAAQoL questionnaire

TAAQOL	Males			Females		
		
	mean (SD)	mean (SD)		mean (SD)	mean (SD)	
	MD	Healthy		MD	Healthy	
	N = 46	N = 1962	d	N = 21	N = 2354	d
Gross Motor	34.6 (26.3)***	88.2 (21.2)	2.5	29.5 (23.6)***	83.7 (24.9)	2.2
Fine Motor	83.1 (20.8)***	97.4 (10.4)	1.4	77.4 (16.9)***	93.8 (16.0)	1.0
Cognitive Functioning	82.7 (24.4)	83.4 (22.0)	0.0	76.5 (29.8)	82.3 (23.4)	0.3
Sleeping	77.6 (32.1)	78.8 (24.1)	0.1	39.9 (36.4)**	69.8 (27.1)	1.1
Pain	69.4 (24.2)*	76.8 (22.9)	0.3	61.3 (33.7)	70.4 (25.0)	0.4
Social Functioning	89.5 (19.5)	84.8 (17.3)	-0.3	91.4 (12.4)**	82.8 (20.7)	-0.4
Daily activities	76.2 (31.4)*	85.8 (22.5)	0.4	65.2 (33.6)*	81.5 (26.5)	0.6
Sexual functioning	86.6 (30.4)	83.6 (27.7)	-0.1	91.9 (15.9)	86.3 (23.6)	-0.2
Vitality	53.6 (24.6)***	68.0 (22.4)	0.6	43.3 (31.6)*	60.4 (24.7)	0.7
Happiness	67.6 (21.5)	65.2 (21.0)	-0.1	60.3 (18.6)	64.1 (22.4)	0.2
Aggressiveness	78.7 (23.8)*	87.5 (17.2)	0.5	76.7 (26.5)	87.9 (16.4)	0.7
Depressive moods	72.3 (24.3)*	81.6 (19.2)	0.5	64.7 (21.9)*	75.0 (21.3)	0.5

One sample t-tests *p < 0.05 **p < 0.01 ***p < 0.001

d = effect size

Higher score represent a better HRQoL

Differences for males were small on the scales of pain and daily activities, moderate on the scales of aggressiveness, depressive moods and vitality, and large for gross and fine motor functioning. Differences for females were moderate on the scales of daily activities, vitality and depressive moods, and large for gross and fine motor functioning and sleeping.

### Influence of type and severity of MD in adults on HRQoL

Significant differences on the TAAQoL (Table 5) between the types of MD were found for fine motor functioning, sleeping, daily activities, vitality and depressive mood. Patients with BMD had higher HRQoL scores on fine motor functioning and sleeping than sarcoglycanopathy patients, and higher scores on daily activities and depressive moods than calpainopathy patients. Although a significant difference was found for vitality with the analysis of variance, the scheffe-procedure just failed to reach significance between groups. Patients with more severe MD had lower scores for fine motor functioning and social functioning (Table 6).

**Table 5:** Mean health-related quality of life scores for adults patients on the TAAQoL according to type of MD

TAAQOL	Types of MD		
	
	mean (SD)	mean (SD)	Mean (SD)
	LGMD2C-F	BMD	LGMD2A
	N = 15	N = 25	N = 23
Gross Motor	36.3 (27.0)	34.5 (27.5)	25.3 (21.7)
Fine Motor	71.7 (18.3)	89.3 (20.0)* X	82.9 (16.6)
Cognitive Functioning	76.7 (27.1)	83.5 (22.7)	79.3 (31.0)
Sleeping	42.5 (41.9)	81.0 (28.7) **X	59.8 (37.8)
Pain	59.2 (34.8)	70.8 (19.3)	66.3 (31.9)
Social Functioning	90.4 (18.0)	93.8 (12.1)	85.3 (22.6)
Daily activities	71.3 (32.7)	84.8 (20.6) *∇	57.9 (38.6)
Sexual functioning	88.5 (20.1)	95.5 (12.5)	78.6 (39.2)
Vitality	39.4 (29.3)	61.0 (19.1)	44.9 (31.9)
Happiness	62.2 (22.9)	72.0 (15.2)	58.7 (23.9)
Aggressiveness	78.5 (24.3)	80.4 (21.6)	73.4 (29.2)
Depressive moods	66.1 (22.2)	80.0 (16.7) *∇	60.1 (28.2)

*p < 0.05 **p < 0.01 ***p < 0.001

∇ = BMD significantly better compared to LGMD2A;

X = BMD significantly better compared to LGMD2C-F

LGMD2C-F = Sarcoglycanopathies; BMD = Becker muscular dystrophy; LGMD2A = Calpaïnopathies

**Table 6:** Mean health-related quality of life scores for adults patients on the TAAQoL according to severity of MD

TAAQOL	Severity	
	
	mean (SD)	mean (SD)
	Less severe	Severe
	N = 25	N = 42
Gross Motor	29.8 (25.7)	35.0 (25.4)
Fine Motor	95.3 (6.8)***	73.0 (20.2)
Cognitive Functioning	79.0 (29.5)	81.8 (24.3)
Sleeping	70.8 (38.9)	62.8 (37.0)
Pain	65.0 (25.3)	68.0 (29.0)
Social Functioning	95.5 (11.2)*	86.9 (19.9)
Daily activities	76.0 (27.3)	70.8 (35.1)
Sexual functioning	91.1 (22.1)	86.5 (29.5)
Vitality	55.0 (26.2)	47.6 (27.6)
Happiness	68.7 (18.4)	63.3 (22.0)
Aggressiveness	73.8 (26.4)	80.7 (23.2)
Depressive moods	74.7 (20.1)	67.1 (25.4)

Student t-tests *p < 0.05 **p < 0.01 ***p < 0.001

Higher scores represent a better HRQoL

## Discussion

This is one of the first studies to describe the health related quality of life (HRQoL) of both children and adults with different muscular dystrophies (MDs) using validated and reliable instruments. The results of the present study indicate that having a MD does negatively influence the HRQoL on several domains, which can be expected because of obvious reasons.

Children (aged 8–11) reported a worse HRQoL on motor functioning and autonomy which can be expected considering the progressive nature of their MD, but they also reported a worse HRQoL on social functioning and emotional functioning as compared to age-related healthy individuals. Especially the latter shows that HRQoL of the patients is affected. The large effect sizes on these two scales underscore the clinical significance of these findings. Surprisingly, both children and adolescents reported a better HRQoL for physical symptoms. Considering the items included on this scale which do not relate to their disease, it may be that children with MD respond emotionally different to such problems than healthy children. Living with their progressive disease possible changed their values, which may be a result of the process of response shift, which has been described in adults with cancer [21]. Response shift implies that the experience with an illness changes the internal standards of patients, resulting in changes in the meaning of their self-evaluation and hence in a possibly different experience of problems.

We did not find cognitive problems for the younger and older children; nor in adults. It is important to notice that children and adolescent do not report more negative emotions compared to their healthy peers. Negative emotions included in the questionnaire are feelings of irritation, or anger. Adult patients, and especially males, however do report negative emotions, e.g. feelings of aggressiveness.

For adult patients HRQoL was lower on several scales as compared to the general population. Pain was reported, only by males, but not associated with type or severity of MD. This finding supports those of Abresch et al [22] who studied the effect of pain on the QoL of individuals with slowly progressive neuromuscular disease (NMD), including a group of LGMD patients. They found that with the exception of adult spinal muscular atrophy, the frequency and severity of pain reported in slowly progressive NMDs was significant.

Within-group differences were studied for the adult patients. For type of MD we found a more favorable HRQoL for BMD patients. Interindividual disease progression variation in BMD is extensive, and several of these patients were only mildly affected. For severity only minor differences were found, possibly explained by BMD patients. Consequently, some confounding of type of MD and severity should be taken into account.

Results of this study show that patients with more severe disease do not necessarily have a worse HRQoL. Only differences on fine motor and social functioning were found. Apparently, severity is not a factor that significantly influences HRQoL. This is in line with findings of Abresch et al [8] who also studied factors that influence HRQoL in patients with neuromuscular diseases. They found that level of disability is not a factor that significantly alters life satisfaction. They postulate that this is because physical functioning has been adequately managed by the patients. Comparable findings are reported by Kohler et al. [23]. They report that quality of life is not correlated with physical impairment.

There are a few limitations to this study. Firstly, the study has a relatively small sample size. Secondly, there is probably a selection bias. All patients who participate in this study were participating already in another study. It is most likely that this has positively influenced the response rate. Thirdly, most assessments of the questionnaire were done by phone. It is not clear how this may have influenced the responses. Fourth, only a restricted number of predictors, namely severity and type of muscular dystrophy, could be investigated, and only in the adult patient population. For example, use of medication such as prednisone or other corticosteroids could also influence the HRQoL outcome. More longitudinal research is needed to trace the patients, at risk for adjustment problems. An interesting area of research would be the coping resources patients use, and preferably the research should be conducted administering questionnaires to patients in person and interviewing them. Furthermore, nearly all of the children participating in this study were boys with Duchenne muscular dystrophy. Therefore, the generalizability of this data to children with other forms of MD and to girls does not seem valid.

Overall, it should be acknowledged that the findings for adults only show a few large effect sizes on especially functional domains (gross and fine motor functioning) and for sleeping for females only. Moderate clinical differences are found for aggressiveness, depressive moods and vitality. Communication about such problems in clinical practice should be encouraged to evaluate in which way the patient is troubled. This can be stimulated by computer-scored individually measurement of HRQoL, in order to inform the physician about the patient's HRQoL [24,25]. The computer output – usually a graphical summary of HRQoL outcomes – assists the physician to focus at the HRQoL domains that correspond with patients needs. Utilizing HRQoL measurement can facilitate patient-physician communication and identifying patients with the greatest needs, so that referring to other health care providers is possible.

## Conclusion

In conclusion, this is one of he first studies to describe the HRQoL of patients with MD using validated and reliable instruments in different age groups. The results of the present study indicate that having MD negatively influences HRQoL. Even though children and adult patients are often considered resilient, HRQoL data show several areas in which patients report difficulties. Clinical significant differences are especially found for younger children. We believe that children and adult patients should be monitored carefully in order to provide additional support such as information, emotional and educational guidance.

## Abbreviations

BMD Becker Muscular Dystrophy

DMD Duchenne Muscular Dystrophy

HRQoL Health Related Quality of Life

LGMD Limb Girdle Muscular Dystrophies

MD Muscular Dystrophy

QoL Quality of Life

TACQoL TNO-AZL Children's Quality of Life questionnaire

TAAQoL TNO-AZL Adult Quality of Life questionnaire

## Competing interests

The author(s) declare that they have no competing interests.

## Authors' contributions

MAG contributed to the analysis and interpretation of data and wrote the manuscript. JB collected the data for this study, and drafted the manuscript. AJK designed the study, contributed to the interpretation of the data and critical revision of the manuscript. All authors read and approved the final version of the manuscript.
